# CHBP induces stronger immunosuppressive CD127^+^ M-MDSC via erythropoietin receptor

**DOI:** 10.1038/s41419-021-03448-7

**Published:** 2021-02-12

**Authors:** Jiawei Li, Guowei Tu, Weitao Zhang, Yi Zhang, Xuepeng Zhang, Yue Qiu, Jiyan Wang, Tianle Sun, Tongyu Zhu, Cheng Yang, Ruiming Rong

**Affiliations:** 1 Department of Urology, Zhongshan Hospital, Fudan University, Shanghai, 200032 China; 2 Shanghai Key Laboratory of Organ Transplantation, Shanghai, 200032 China; 3 Department of Critical Care Medicine, Zhongshan Hospital, Fudan University, Shanghai, 200032 China; 4 Zhongshan Hospital Institute of Clinical Science, Zhongshan Hospital, Fudan University, Shanghai, 200032 China; 5 Department of Urology, Shanghai Public Health Clinical Center, Shanghai, 201508 China; 6 Jiangsu Key Laboratory of Infection and Immunity, Institutes of Biology and Medical Sciences, Soochow University, Suzhou, 215006 China; 7 Shanghai Public Health Clinical Center, Shanghai, 201508 China; 8 Zhangjiang Institute of Fudan University, Shanghai, 201203 China; 9 Department of Transfusion, Zhongshan Hospital, Fudan University, Shanghai, 200032 China

**Keywords:** Allotransplantation, Inflammatory diseases

## Abstract

Erythropoietin (EPO) is not only an erythropoiesis hormone but also an immune-regulatory cytokine. The receptors of EPO (EPOR)_2_ and tissue-protective receptor (TPR), mediate EPO’s immune regulation. Our group firstly reported a non-erythropoietic peptide derivant of EPO, cyclic helix B peptide (CHBP), which could inhibit macrophages inflammation and dendritic cells (DCs) maturation. As a kind of innate immune regulatory cell, myeloid-derived suppressor cells (MDSCs) share a common myeloid progenitor with macrophages and DCs. In this study, we investigated the effects on MDSCs differentiation and immunosuppressive function via CHBP induction. CHBP promoted MDSCs differentiate toward M-MDSCs with enhanced immunosuppressive capability. Infusion of CHBP-induced M-MDSCs significantly prolonged murine skin allograft survival compared to its counterpart without CHBP stimulation. In addition, we found CHBP increased the proportion of CD11b^+^Ly6G^−^Ly6C^high^ CD127^+^ M-MDSCs, which exerted a stronger immunosuppressive function compared to CD11b^+^Ly6G^−^Ly6C^high^ CD127^−^ M-MDSCs. In CHBP induced M-MDSCs, we found that EPOR downstream signal proteins Jak2 and STAT3 were upregulated, which had a strong relationship with MDSC function. In addition, CHBP upregulated GATA-binding protein 3 (GATA-3) protein translation level, which was an upstream signal of CD127 and regulator of STAT3. These effects of CHBP could be reversed if *Epor* was deficient. Our novel findings identified a new subset of M-MDSCs with better immunosuppressive capability, which was induced by the EPOR-mediated Jak2/GATA3/STAT3 pathway. These results are beneficial for CHBP clinical translation and MDSC cell therapy in the future.

## Introduction

Erythropoietin (EPO), as an endogenous protein for red blood cell production, has been discovered for over a century. Although the name of EPO refers mainly to the function of erythropoietic hormone, this molecule also exerts important effects as a cytokine and growth factor that affect multiple organs, including the immune system. The unique tissue-protective receptor (TPR), which is mainly consisted of EPO receptor (EPOR) and β common receptor, mediates EPO-involved anti-inflammation, anti-apoptosis, and immune regulation functions^1^. Thus, EPO is a multifaceted protein due to its nonerythropoietic effects^2^. However, the very high dosage required to achieve that goal would cause unfavorable side effects, including hypertension and thrombosis. A series of EPO derivant have been developed including carbamoylated EPO and peptides^3^. Our group firstly synthesized and reported a novel proteolysis-resistant cyclic helix B peptide (CHBP) with improved metabolic stability and tissue-protective potency^4^.

Theoretically, CHBP inherits EPO-mediated immune regulation effects because of they share TPR. In fact, CHBP reveals excellent cell, tissue, and organ protective property such as mesenchymal stem cell, kidney, and heart^4–6^. Cravedi et al. found that EPO/EPOR signal inhibited conventional T-cell proliferation in vitro via tyrosine phosphatase SHP-1–dependent uncoupling of IL-2Rβ signaling. Conversely, EPO-initiated signals facilitated Treg proliferation by augmenting IL-2Rγ signaling and maintaining constitutively quenched IL-2Rβ signaling. Furthermore, EPO prolonged heart allograft survival, whereas pharmacologic downregulation of kidney-derived EPO abrogated kidney allograft acceptance^7^. These results suggest manipulating the EPO/EPOR signaling axis could be exploited to prevent transplant rejection. In our previous study, we found CHBP administration significantly reduced kidney allograft acute rejection in a rat model. CHBP inhibited dendritic cell (DC) maturation via Jak2/STAT3 pathway, which was a classical downstream signal upon EPOR^8^. Taken into these two types of research together, EPOR signal, either stimulated by EPO or CHBP, can modulate both innate and adaptive immunity^2^.

Targeting adaptive immune response is not the only focus when prevention and treatment of allograft rejection. Innate immune response provocation and regulation are to be of importance in the pathological process^9,10^. As a kind of innate immune cell, myeloid-derived suppressor cells (MDSCs) share a common myeloid progenitor with DC. MDSCs are not a terminally differentiated population of cells. In mice, MDSCs are defined as CD11b^+^Gr1^+^ cells. Usually, MDSCs can be divided into two major groups, which can be identified by a combination of specific markers. Granulocytic MDSCs (G-MDSCs) are defined as CD11b^+^Ly6G^+^Ly6C^low^ cell and monocytic MDSCs (M-MDSCs) are defined as CD11b^+^Ly6G^−^Ly6C^high^ cells in mice. The main characteristic of MDSCs is immunosuppression in terms of suppression of T- and B-cell proliferation, induction of T-cell apoptosis, inhibition of DC development and macrophages cytokine production, impairment of the effect of natural killer cells on alloantigens and promotion of regulatory T cells (Tregs)^11^. It is reported that MDSCs reduced allograft rejection as well as induced tolerance in transplantation^12–14^, and M-MDSCs are more suppressive than G-MDSCs on a per-cell basis^15^. Therefore, it might be more effective to induce more M-MDSCs in vivo during tolerance induction or prevention of allograft rejection. In addition, whether the EPOR signal regulates MDSCs differentiation and function is unknown.

In the current study, we found CHBP induced more M-MDSCs rather than G-MDSCs. The M-MDSCs induced by CHBP showed a more potent immunosuppressive capability. Infusion of CHBP-induced M-MDSCs ameliorated skin allograft rejection and prolonged allograft survival. Furthermore, we identified a new M-MDSCs subset with CD127 positive, which revealed a strong immunosuppressive function than CD127^−^ M-MDSCs. CHBP significantly increased this CD127^+^ M-MDSCs subset proportion via EPOR.

## Results

### CHBP induced M-MDSCs differentiation and enhanced M-MDSCs immunosuppressive function

**Fig. 1:**
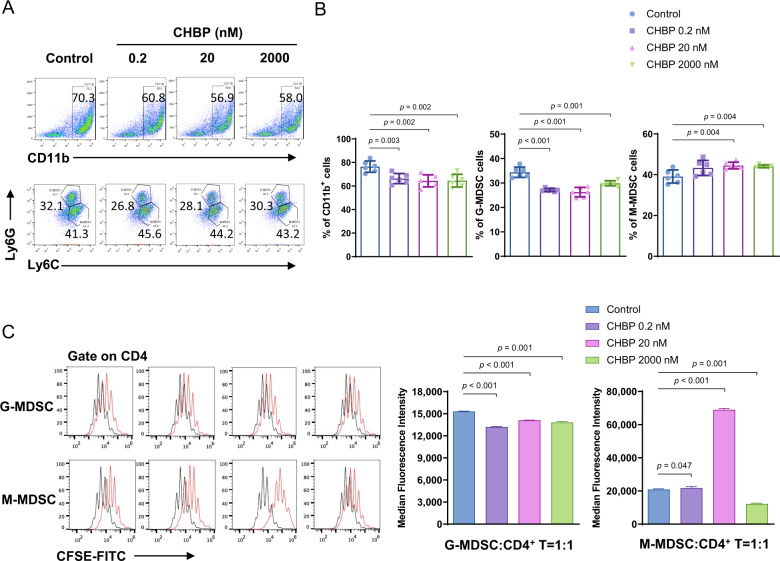
CHBP induced M-MDSCs differentiation and enhanced M-MDSCs immunosuppressive function. **A** Detection of CD11b^+^ myeloid cells, CD11b^+^Ly6G^−^Ly6C^high^ M-MDSCs, and CD11b^+^Ly6G^+^Ly6C^low^ G-MDSCs by flow cytometry. **B** Quantification analysis of CD11b^+^ myeloid cells, M-MDSCs and G-MDSCs. **C** T-cell proliferation detected by CFSE. The ratio of G-MDSCs, M-MDSCs, and T cells was 1:1.

To understand whether the presence of CHBP directly alters the development of MDSCs, we detected the CD11b^+^ cells differentiation, numbers, phenotypes, and immunosuppressive function of bone marrow cells which were cultured with granulocyte-macrophage colony-stimulating factor (GM-CSF) or GM-CSF + CHBP for 7 days. We added CHBP with different dosages (0.2, 20, and 2000 nM) during MDSCs induction in vitro. Firstly, we found that despite CHBP decreased CD11b^+^ cells proportion and number, the proportion of M-MDSCs was significantly increased with a reduction of G-MDSCs ratio (Figs. 1A, B and S1), suggesting that CHBP regulates MDSCs differentiation toward M-MDSCs.

To determine the immunosuppressive effect of GM-CSF + CHBP induced G-MDSC and M-MDSC on T-cell activation, we detected cell proliferation productions of T cells stimulated by ConA in the presence of the induced MDSCs at different CHBP doses. The addition of GM-CSF induced M-MDSCs markedly inhibited the proliferative response of CD4^+^ T cells comparing to G-MDSCs (Fig. 1C). However, GM-CSF + CHBP-induced G-MDSC showed lessened suppressive function, but GM-CSF + CHBP induced M-MDSC possessed stronger immunosuppressive ability on T-cell proliferation than GM-CSF-induced M-MDSCs, especially in the 20 nM CHBP group (Fig. 1C). Thus, CHBP could increase M-MDSCs proportion and enhance the immunosuppressive function of GM-CSF-induced M-MDSCs.

### Infusion of CHBP-induced M-MDSCs prolonged skin allograft survival

**Fig. 2:**
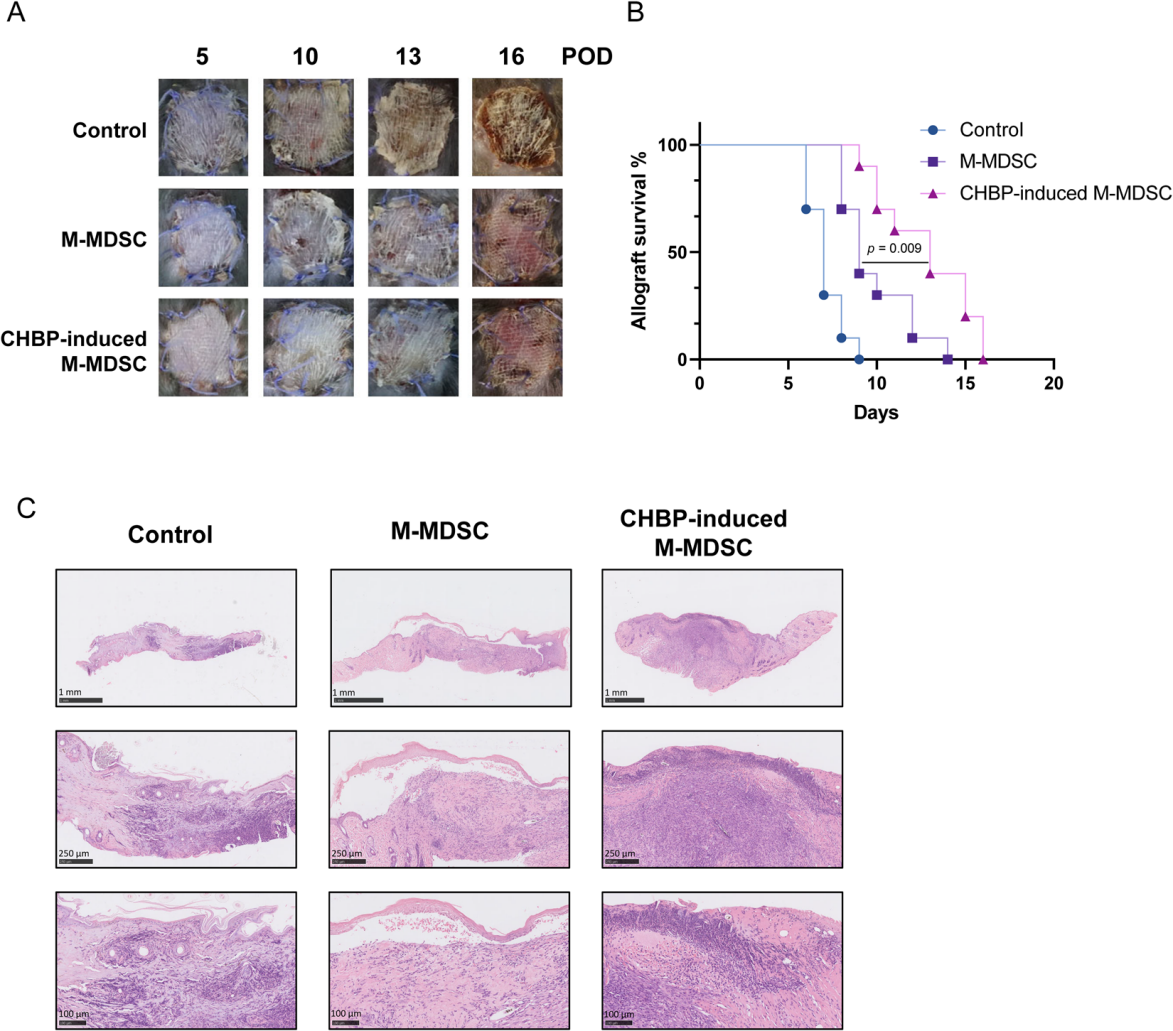
Infusion of CHBP-induced M-MDSCs prolonged skin allograft survival. **A** Skin pictures at different days post transplantation. **B** The survival rates. **C** H&E staining of the skin (POD 14). Scale bars: 1st row 1 mm, 2nd row 250 μm, and 3rd row 100 μm.

Since CHBP-induced M-MDSCs exerted enhanced immunosuppressive function, we employed the BALB/c (H2-D) alloskin-grafted B6 (H2-B) mouse model and infused these cells into the recipient mice. We took pictures at different times to evaluate the rejection degree (Fig. 2A). M-MDSCs with CHBP treatment significantly extended the skin allograft survival compared to the M-MDSCs without CHBP induction (Fig. 2B). The rejection and tissue scores were also reduced in the CHBP-induced M-MDSC group (Fig. 2C). Thus, GM-CSF + CHBP-induced M-MDSC could increase the allograft survival and promote tissue healing.

### Infusion of CHBP-induced M-MDSCs decreased CD4^+^, CD8^+^ T cells and increased Tregs in peripheral and skin

**Fig. 3:**
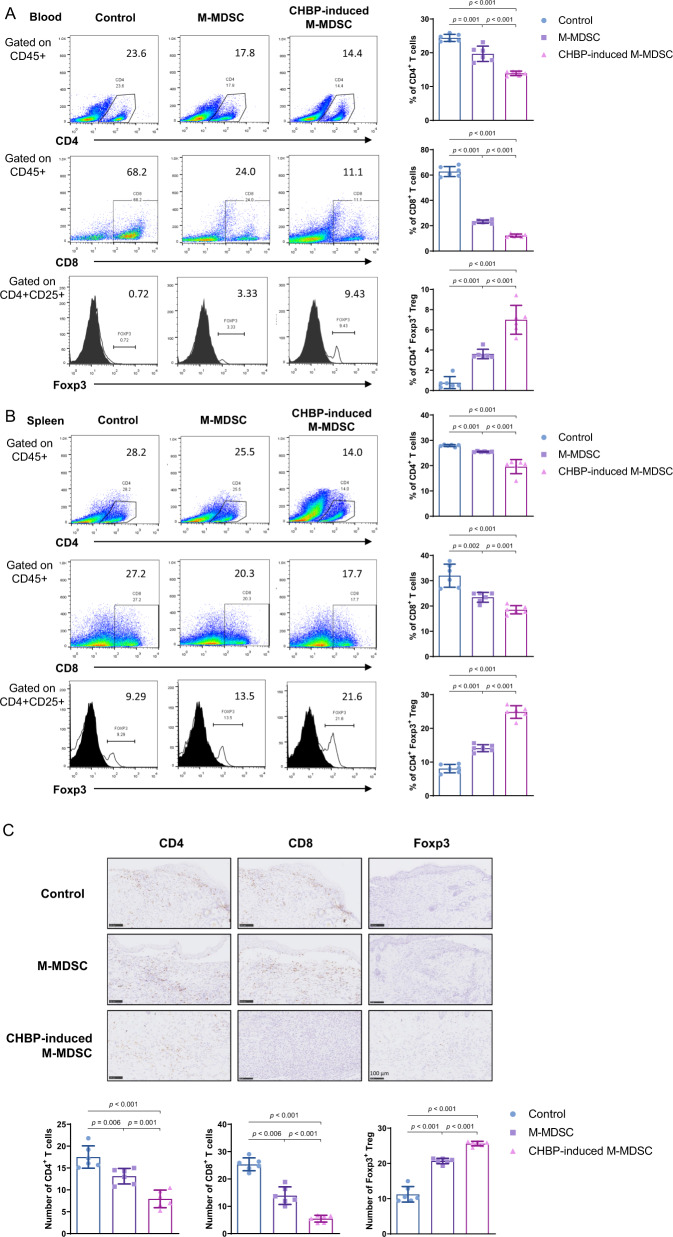
Infusion of CHBP-induced M-MDSCs decreased CD4^+^, CD8^+^ T cells, and increased Tregs in peripheral and skin. **A** Detection of the proportion of CD4^+^, CD8^+^ T cells, and Foxp3^+^ Tregs in the blood. **B** Detection the proportion of CD4^+^, CD8^+^ T cells, and Foxp3^+^ Tregs in the spleen. **C** CD4, CD8, and Foxp3 immunohistochemistry staining in the alloskin with semi-quantification analysis (POD 14). Scale bar: 100 μm.

To investigate the influence on T cell number in the peripheral and infiltration in the skin, we examined the CD4^+^, CD8^+^ T cells, and Tregs in the blood, spleen, and skin. Flow cytometry analysis demonstrated the infusion of CHBP-induced M-MDSCs decreased the proportion of CD4^+^, CD8^+^ T cells and increased Tregs in the blood (Fig. 3A), as well as in the spleen (Fig. 3B). Similar results were observed in the skin (Fig. S2b). These results suggested that infusion of CHBP-induced M-MDSCs ameliorated effector T cells response and favorited Tregs. However, the percentage of CD3^+^ cells in blood showed no significant differences between groups (Fig. S2a), which suggested that neither M-MDSC nor CHBP-induced M-MDSC had an influence on circulating T cells. To determine whether CHBP could induce activated T cells into Tregs in vitro, we performed the Transwell test and found that M-MDSC induced by CHBP induced more Tregs than their counterparts without CHBP stimulation (Fig. S2c).

### The effects of CHBP on MDSCs were dependent on EPOR

**Fig. 4:**
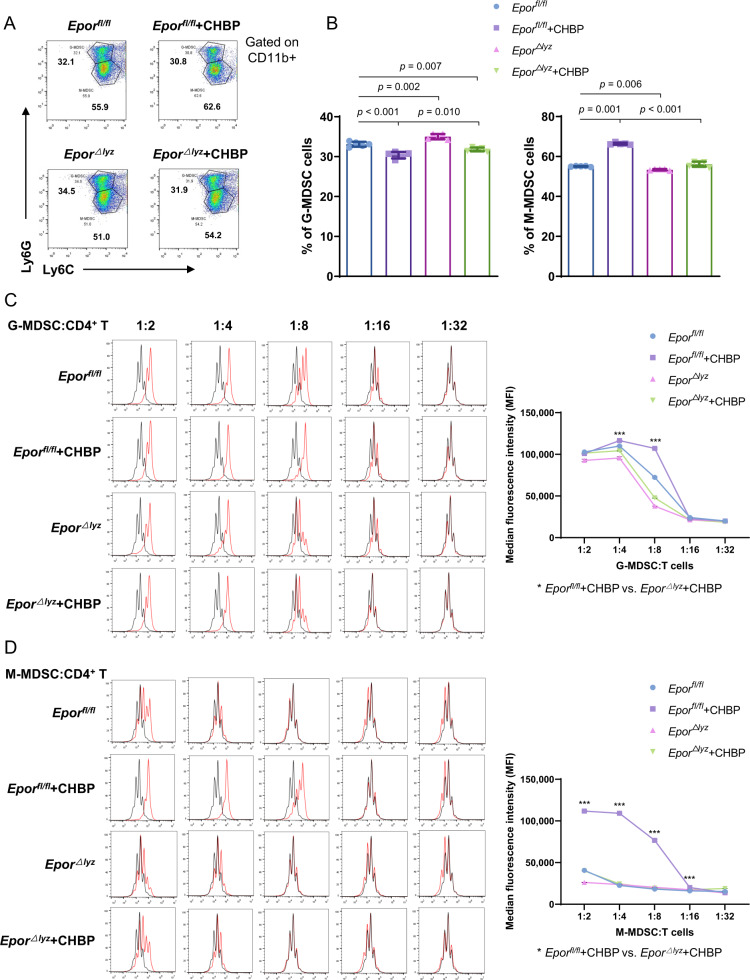

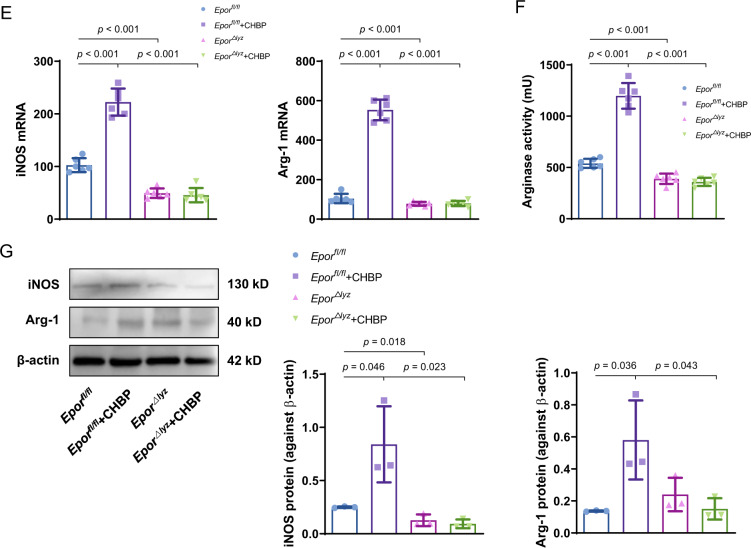
The effects of CHBP on MDSCs were dependent on EPOR. **A** Detection of CD11b^+^Ly6G^−^Ly6C^high^ M-MDSCs and CD11b^+^Ly6G^+^Ly6C^low^ G-MDSCs by flow cytometry. **B** Quantification analysis of the G-MDSCs and M-MDSCs proportions. **C** T-cell proliferation detected by CFSE. The ratios of G-MDSCs and T cells were 1:2, 1:4, 1:8, 1:16, and 1:32. **D** T-cell proliferation detected by CFSE. The ratios of M-MDSCs and T cells were 1:2, 1:4, 1:8, 1:16, and 1:32. **E** The expressions of iNOS and Arg-1 by qPCR. **F** The arginase-1 activity. **G** The protein expressions of iNOS and Arg-1 by western blot.

As we mentioned in the introduction, CHBP shares EPOR with EPO, on which their immune regulation functions are dependent. In this study, we generated *Epor*^*△lyz*^ mice, whose myeloid-derived cells lack EPOR. In in vitro experiments, the MDSCs deficient of EPOR had a slight influence on differentiation in terms of more G-MDSCs and fewer M-MDSCs. In CHBP treatment groups, deficiency of EPOR abolished CHBP effects, especially on M-MDSCs induction (Fig. 4A, B). Next, we performed a T-cell proliferation assay to examine the immunosuppressive function of G-MDSCs and M-MDSCs. We use different ratios between MDSCs and T cells. Although T cell proliferation was not inhibited by G-MDSCs at a 1:1 ratio in the above result (Fig. 1D), CHBP-induced G-MDSCs still acquired enhanced immunosuppressive function. Knockout of *Epor* in G-MDSCs reduced CHBP effect (Fig. 4C). The results of M-MDSCs were amazing. CHBP increased 1.1–4.5 folds immunosuppressive function of M-MDSCs at 1:16, 1:8, 1:4, and 1:2, respectively (*Epor*^*fl/fl*^ + CHBP vs. *Epor*^*△lyz*^ + CHBP, Fig. 4D).

To confirm whether the inhibitory function of CHBP induced M-MDSCs, we detected the mRNA and protein expressions of inducible NO synthase (iNOS) and arginase-1 (Arg-1) in sorted CD11b^+^Ly6G^−^Ly6C^high^ cells. Both of iNOS and Arg-1 were significantly increased by CHBP in *Epor*^*fl/fl*^ M-MDSCs, but reversed in *Epor*^*△lyz*^ M-MDSCs (Fig. 4E, G). A similar result was observed in Arg-1 activity (Fig. 4F). These results indicate that CHBP enhances M-MDSCs immunosuppressive function through EPOR.

### CD127^+^ M-MDSCs induced by CHBP exerted stronger immunosuppressive function

**Fig. 5:**
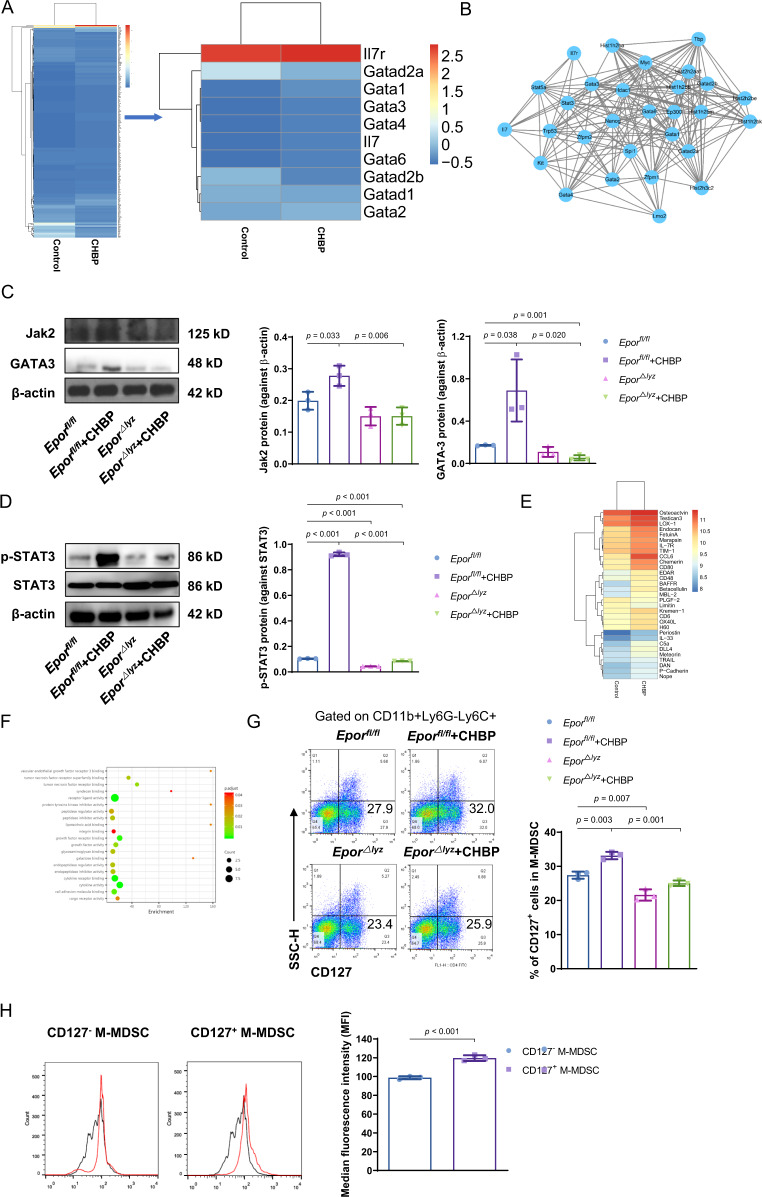
CD127^+^ M-MDSCs induced by CHBP exerted stronger immunosuppressive function. **A** RNA-seq detected the different RNA expressions in M-MDSCs with or without CHBP treatment in vitro. **B** The String analysis of mRNA sequencing. **C** The Jak2 and GATA3 protein expression by western blot. **D** The p-STAT3 and STAT3 protein expression by western blot. **E** Protein array compared the difference in M-MDSCs with or without CHBP treatment in vitro. **F** The molecular function enrichment of protein array. **G** Detection and quantification of the CD127-positive cells in M-MDSCs by flow cytometry. **H** T-cell proliferation detected by CFSE. The ratio of G-MDSCs and T cells was 1:1.

To further investigate the mechanism that CHBP enhanced M-MDSCs immunosuppressive function, we analyzed the mRNA differences between the two groups of M-MDSCs with or without CHBP treatment using mRNA sequencing (Fig. 5A). We found that the mRNA expression of GATA-binding protein 3 (GATA3) was slightly increased with an IL-7R increasing by CHBP. String analysis showed the protein–protein interaction and we found that there was a strong connection between GATA3, STAT3, and IL-7R (Figs. S3a and 5B). Hoyler et al. reported that GATA3 controlled IL-7R expression^16^, we speculate CHBP regulates GATA3 expression and then induces IL-7R expression. Therefore, we further examined the Jak2/GATA3/STAT3 protein level in M-MDSCs. We found that Jak2, GATA3, p-STAT3, and STAT3 protein level was significantly increased by CHBP in *Epor*^*fl/fl*^ M-MDSCs, and the effect was abolished when M-MDSCs deficient of *Epor* (Fig. 5C, D). Next, we extracted membrane proteins of M-MDSCs and performed a protein array to further check IL-7R protein level (Figs. S2b and 5E). Molecular function enrichment showed that CHBP-induced MDSCs had intense function with receptor-ligand and cytokine receptor activity (Fig. 5F). In addition, biological process and KEGG pathway also demonstrated CHBP induced MDSCs have a close relationship with cell proliferation and cytokine–cytokine receptor interaction (Fig. S3c, d). GO and KEGG pathway analyses revealed that CHBP increased M-MDSCs cell proliferation and receptor activity. IL-7R, also named as CD127, is a membrane receptor protein. We examined the proportion of CD127^+^ M-MDSCs using flow cytometry (Fig. 5G). These results demonstrated that CHBP significantly upregulated CD127^+^ M-MDSCs in total M-MDSCs, whose effect was dependent on EPOR. At last, we sorted these CD127^+^ M-MDSCs from CHBP-induced M-MDSCs and compared their immunosuppressive function to CD127^−^ M-MDSCs using CFSE-labeled T cells proliferation assay. The results showed CD127^+^ M-MDSCs had prominent stronger immunosuppressive capacity than CD127^−^ M-MDSCs (Fig. 5H). These results suggest that CHBP enhances M-MDSCs immunosuppressive function might be attributed to the induction of CD127^+^ M-MDSCs.

## Discussion

In recent years, numerous studies have shown that EPO acts far beyond erythropoiesis. The immune regulatory function of EPO endows EPO and its derivant-based drug development more application possibilities^17^. There are two kinds of key receptors combined with EPO (EPOR)_2_ and TPR. (EPOR)_2_ and TPR are expressed on a variety of immune cells, such as macrophages, DCs, mast cells, and lymphocytes^18^. An increasing body of evidence demonstrates that EPO and its derivatives can directly affect the manner by which immune cells exert their immunoregulatory effects. For instance, our group found that EPO ameliorated acute kidney injury by reducing macrophage infiltration and promoting M2 phenotype polarization in vivo^19^. EPOR is also expressed on DC^20^. We previously demonstrated that CHBP could ameliorate acute rejection in a rat kidney transplantation model via inhibition of DC maturation^8^. This study confirmed that CHBP-regulates MDSCs differentiation and function via EPOR signal. This is the first report about EPOR-mediated immune regulation on MDSCs.

It is encouraging that CHBP promotes MDSCs to differentiate toward M-MDSCs and empowers M-MDSCs with stronger immunosuppressive function than G-MDSCs. Although both G-MDSCs and M-MDSCs have the immunosuppressive ability, the strength and mechanisms are different. It is now accepted that M-MDSCs are more suppressive than G-MDSCs on a per-cell basis^15^. Therefore, CHBP demonstrates a bright prospect clinical translation in M-MDSCs-based cell therapy. To be in fact, our data showed CHBP also enhanced G-MDSCs immunosuppressive function according to Fig. 4C. It seems that there were opposite results in Figs. 1D and 4C, our explanation is the difference ratio between G-MDSCs and T cells.

The most important finding in our research is that CHBP induced CD127^+^ M-MDSCs. CD127 is the α chain of IL-7R. It is well-known that low expression of CD127 is a cell surface marker of human Tregs which could be instead of intracellular Foxp3. So, the CD4^+^CD25^+^CD127^low^ T cells represent human Tregs. However, some recent studies demonstrated an opposite expression pattern of CD127 in innate immune regulatory cells. In 2016, Björklund et al.^21^ found a heterogeneity of CD127 in innate lymphoid cells (ILCs) using single-cell RNA sequencing. Next, Fan’s group identified a novel subset of ILCs, Lin^−^CD45^+^CD127^+^IL-10^+^ ILCreg. The ILCreg secrets IL-10 and TGF-β1, with immunosuppressive function^22^. Autoimmune regulator (AIRE) plays a key role in central and periphery immune tolerance. A recent study confirmed these AIRE expression cells outside the thymus are CD127^+^CCR7^+^ PD-L1^+^ DCs^23^. Taken these studies together, CD127 might be an important cell surface marker of some innate immune regulatory cells. Our results for the first time identified a new subset of MDSCs, CD11b^+^ Ly6G^−^Ly6C^high^CD127^+^ M-MDSCs. The M-MDSCs with CD127 expression reveal more immunosuppressive ability than those without CD127 expression. However, the mechanism is still unknown. Does CD127 directly combine with target immune cells or indirectly initiate other signal transductions? This question might be answered in a future experiments.

We elucidated that GATA3 is the key transcription factor of which CHBP induced CD127 expression in M-MDSCs. GATA3 is a double zinc-finger transcription factor that is required for the effector fate decision of Th2 cells^24,25^. GATA3 integrates diverse upstream signals to control target gene expression and cellular functions^26^. Hoyler et al. demonstrated that GATA3 is essential for ILC2 fate decisions, whose cell surface marker contains CD127^16^. CD127 expression was significantly lower in GATA3-deficient CD8^+^ T cells than in wild-type (WT) counterparts. In addition, compared to WT CD8^+^ T cells, GATA3-deficient CD8^+^ T cells were defective in responding to IL-7-promoted T-cell survival in vitro. As a matter of fact, GATA3 could bind to the *Il7r* locus^27^. Here, we found that GATA3 protein translation could be up-regulated upon EPOR signal stimulation rather than its mRNA transcription. Then the increased GATA3 might promote more CD127 expression in M-MDSCs. STAT3 is probably one of the main transcription factors that regulate MDSC function. Recent works have highlighted the importance of signaling pathways downstream of STAT3 that are responsible for MDSC differentiation in tumor models^28^. Besides *Il7r*, GATA3 also bounds to the −1710 to −1530 region of STAT3 promoter and repressed its transcription^29^. Therefore, GATA3 might play a central role in the new CD127^+^ M-MDSCs subset differentiation and function.

In conclusion, the EPO derivant peptide CHBP induces M-MDSCs differentiation and enhanced M-MDSCs immunosuppressive function via EPOR-mediated Jak2/GATA3/STAT3 pathway. Infusion of these induced M-MDSCs prolongs skin allograft survival. CHBP induces more CD11b^+^Ly6G^−^Ly6C^high^CD127^+^ M-MDSCs, which have more potent immunosuppressive function compared to CD127^−^ counterparts. Our novel findings identified a new subset of M-MDSCs with the better immunosuppressive capability and demonstrated EPOR-mediated Jak2/GATA3/STAT3 pathway. These results are benefit for CHBP clinical translation in the future.

## Materials and methods

### Animal strains

C57BL/6 (B6) and BALB/c mice were purchased from the Slac. Company (Shanghai, China). The myeloid-specific EPOR conditional knockout mice (Lyz-Cre/Epor^loxp/loxp^) under C57BL/6 genetic background were obtained by crossing Epor^loxp/loxp^ mice with mice expressing Cre recombinase under the control of the Lysozyme promoter (Lyz-Cre). Epor^loxp/loxp^ littermates served as the WT control. Lyz-Cre mice were kindly provided by Professor Yong Zhao from the Institute of Zoology, Chinese Academy of Sciences (Beijing, China). Epor^loxp/loxp^ mice were generated with Cyagen Biosciences Inc. (Shanghai, China). Mice were used between 6 and 8 weeks of age. Experimental protocols were approved by the Animal Ethics Committee of Zhongshan Hospital Fudan University (Shanghai, China).

### Generation of Epor^loxp/loxp^ mice

Epor floxed mice were generated by Cyagen Biosciences. Briefly, the *Epor* gene was located on chromosome 9 in mice. The linearized vector was subsequently delivered to ES cells (C57BL/6) via electroporation, followed by drug selection, PCR screening, and Southern Blot confirmation. After gaining 94 drug-resistant clones, we have confirmed 12 potentially targeted clones, 6 of which were expanded for Southern Blotting. After confirming correctly targeted ES clones via Southern Blotting, we selected some clones for blastocyst microinjection, followed by chimera production. Founders were confirmed as germline-transmitted via crossbreeding with WT. In the end, four male and four female F1 heterozygous mutant mice confirmed as the final deliverables for this project.

### Mice skin transplantation model

Recipient B6 mice were injected with i.v. 2 × 10^6^ MDSC or CHBP induced MDSC and the matched controls were given equal volumes of 0.9% saline solution. Full-thickness tail skin tissue of BALB/c mice was grafted on the dorsal part of the C57BL/6 (B6) recipients on day 0, according to the procedure as described previously^30^. Photographs were taken daily with a digital camera until the graft was rejected completely. Fourteen days later, the percentages of CD4^+^, CD8^+^, and CD4^+^CD25^+^Foxp3^+^ cells in the peripheral blood and spleens were detected by flow cytometry.

### Induction of MDSCs from bone marrow cells

Bone marrow cells from tibias and femurs of C57BL/6 and *Epor*^*△lyz*^ mice (6–8 weeks) were flushed with PBS and the red blood cells were lysed by RBC lysis buffer (BD Biosciences, CA, USA). Totally, 2 × 10^6^ non-adherent bone marrow cells were cultured with 50 ng/ml GM-CSF (PeproTech, RH, USA) or 50 ng/ml GM-CSF + 20 μM CHBP, respectively, in 6 cm dishes (Corning, USA) in 3 ml of complete RPMI 1640 medium (containing 10% FBS, 1% MEM nonessential amino acids (NEAA) solution, 1% sodium pyruvate, 1% streptomycin and penicillin, and 2 μl 2-Mercaptoethanol) (RPMI 1640 medium, FBS, MEM NEAA, sodium pyruvate, and streptomycin and penicillin were purchased from Gibco, NY, USA; 2-Mercaptoethanol was purchased from Sigma-Aldrich, St. Louis, USA) at 37 °C, 5% CO_2_ for 7 days.

### Cell staining and flow cytometry

After 7-day induction, induced MDSCs were tested or sorted on Beckman Coulter MoFlo Astrios^EQ^ (Beckman Coulter, CA, USA) or FACS AriaIII (BD Biosciences, San Diego, CA, USA) by using mAb CD11b (clone: M1/70), Ly-6G (clone: 1A8), Ly-6C (clone: AL-21), and CD127 (clone: A7R34) (all antibodies were purchased from BD Biosciences or eBioscience company). Flow cytometry verified that all the isolated MDSCs yielded a pure population of more than 90%. The sorted MDSCs were stored in PBS + 10% FBS for further experiments.

Splenocytes and peripheral blood mononuclear cells were obtained from spleens and peripheral blood of mice by Ficoll density gradient centrifugation. Cells were tested by using mAb CD45 (clone: 30-F11), CD3 (clone: 17A2), CD4 (clone: GK1.5), CD8 (clone: 53-6.7), and Foxp3 (clone: FJK-165) (all antibodies were purchased from BD Biosciences, eBioscience or Biolegend company) on BD FACSCallibur (BD Biosciences, San Diego, CA, USA) and analyzed by Flow JoX software.

### T-cell proliferation assay

A single-cell suspension was prepared from spleens of C57BL/6 (B6). Naïve T cells were isolated from mononuclear cells by magnetic-activated cell sorting according to the manufacturer’s instructions (Miltenyi Biotec, Auburn, CA, USA). Cells were labeled with 2 μM CFSE (Invitrogen) for 5 min in PBS at 37 °C and washed twice with PBS. The labeled cells were then stimulated with ConA (eBioscience) (2 μg/ml) in the presence of different doses of MDSCs as indicated for 72 h. The cell proliferation was then determined by flow cytometry after staining with anti-CD4 antibody.

### Real-time quantitative PCR (qPCR)

Total RNA isolation was performed using TRIzol reagent according to the manufacturer’s instructions (Invitrogen). The quality and integrity of RNA were evaluated via A260/A280 ratio and 18s/28s band by agarose electrophoresis. Then total RNA was reversed to the first-strand cDNA using RevertAid First Strand cDNA Synthesis Kit (Thermo Fisher Scientific, Inc.). RT-qPCR was performed in duplicate using All-in-One^™^ qPCR Mix (GeneCopoeia, Inc., Maryland, USA). An Eppendorf Mastercycler Realplex PCR system was used for quantitative PCR under the following conditions: initial denaturation was performed at 95 °C for 10 min, followed by 40 cycles of denaturation at 95 °C for 10 s, annealing at 60 °C for 20 s and extension at 72 °C for 15 s. GAPDH was used as an internal control to normalize differences in the amount of total RNA in each sample. The threshold cycle (Ct) values were analyzed using the comparative Ct (−ΔCt) method. The expression level of target genes was obtained by normalizing to the endogenous reference and relative to control. Primer sequences are listed in Table S1.

### Arginase-1 activity

A total of 1 × 10^6^ cells were mixed with 100 µl of 0.1% Triton X-100. After 30 min of incubation on a shaker, 100 μl of 25 mM Tris-HCL and 20 ul of 10 mM MnCl_2_ were added to the sample. The arginase was then activated by heating the sample for 10 min at 56 °C, and arginine hydrolysis was conducted by incubating the sample with 100 μl of 0.5 M l-arginine (pH 9.7) at 37 °C for 60–120 min. The reaction was stopped with 900 ul of H_2_SO_4_ (96%)/H_3_PO_4_ (85%)/H_2_O, and the sample was mixed with 40 μl of 9% isonitrosopropiophenone. Absorbance was read at 540 nm in the microplate reader. All samples were read in triplicate.

### Western blot

Twenty micrograms of proteins from the MDSCs homogenates were separated on 15% or 10% (wt/vol) polyacrylamide denaturing gels and electro-blotted onto polyvinylidene fluoride membranes. The primary antibodies used were anti-iNOS (1:1000, abcam, USA), Arg-1 (1:1000, Cell Signaling Technology, Danvers, MA, USA), Jak2 (1:1000, Cell Signaling Technology), p-STAT3 (1:1000, Cell Signaling Technology), STAT3 (1:1000, Cell Signaling Technology) and GATA3 (1:1000, Cell Signaling Technology). The semiquantitative analysis (AlphaView Software 3.3, Cell Biosciences, Inc.) results were expressed as the optical volume densities (OD × mm^2^) normalized to GAPDH (1:1000 dilution, Cell Signaling Technology) or β-actin (1:10,000 dilution, Abcam, Cambridge, UK).

### HE staining and pathological assessment

Mice skin tissues were fixed with 10% formalin and bedded with paraffin and were cut into 4 μm thick slices. After dewaxing, HE staining was performed. HE staining was observed under microscopy at 200× to evaluate tissue damage.

Harvested mice skin tissues were fixed overnight in 10% formalin, embedded in paraffin, and cut into 4 μm sections. Sections for CD4, CD8, and Foxp3 staining were incubated with 3% hydrogen peroxide to block endogenous peroxidase for 20 min at 37 °C, and Ags were retrieved using a high-pressure method in citrate buffer. The sections were incubated with an anti-CD4 mAb (1:5000; ab183685, Abcam), an anti-CD8 mAb (1:2000; ab217344, Abcam) or anti-Foxp3 mAb (1:100; 12653, Cell Signaling Technology) at 4 °C overnight. The sections with mice CD4, CD8, or Foxp3 Abs were rinsed three times in TBST and incubated with an HRP-conjugated rabbit anti-goat IgG H&L (1:2000; ab205718, Abcam) at room temperature for 45 min. DAB was added for a coloration for 5 min immunoreactivity was analyzed by image-pro plus.

### RNA sequencing

GM-CSF and GM-CSF + CHBP induced MDSC RNA were extracted with Trizol (15596-026, Invitrogen). RNA sequencing was employed and single-end 75-bp-length reads were generated. Data were aligned to the mouse genome (mm9 version) with the TopHat2 algorithm. HT-seq and DESeq algorithms were used in order to measure gene expression and identify differential expression between the two groups of cells. Genes with a *P* value ≤ 0.05 and fold change ≥ 1.5 or ≤−1.5 were considered to be upregulated and downregulated, respectively. Gene ontology analysis, pathway annotation, transcription factor enrichment, and comparison with various immunological and oncogenic gene signatures were performed with the use of DAVID knowledge base, Ingenuity Pathway Analysis software, and Molecular Signature Database from Broad Institute.

### Protein array

Membrane proteins of GM-CSF and GM-CSF + CHBP induced MDSC were measured with an AAM-CYT-7 RayBio Mouse Cytokine Antibody Array. Differential expression proteins with a *P* value ≤ 0.05 and fold change ≥ 1.2 or ≤0.83 were considered to be upregulated and downregulated, respectively. Gene ontology analysis, pathway annotation, transcription factor enrichment, and comparison with various immunological and oncogenic gene signatures were performed with the use of the DAVID knowledge base.

### Statistics

Data were analyzed using GraphPad Prism 8 software or R language. Packages such as Limma, Annotation, ggplot2, enrichGo were used to analyze the RNA-sequencing and array results. Quantitative variables were analyzed by one-way ANOVA (among three or more groups), two-tailed independent *t* test (between two groups) and expressed as means ± standard deviation (S.D.). *P* < 0.05 was considered statistically significant.

## Supplementary information

Supplementary Figure Legends

Figure S1

Figure S2

Figure S3

Table S1

## Data Availability

Raw data files for RNA-seq of MDSCs can be found at NCBI Gene Expression Omnibus Accession number: GEO: GSE160822.
