# Supplementary material for: CHBP induces stronger immunosuppressive CD127+ M-MDSC via erythropoietin receptor
Source: Cell Death Dis. 2021 Feb 12;12(2):177. doi: 10.1038/s41419-021-03448-7 (PMC7881243; doi:10.1038/s41419-021-03448-7)
Supplement: Supplementary file 1 — Supplementary Figure Legends [file 41419_2021_3448_MOESM1_ESM.docx]

**Supplementary Figure Legends**

**Figure S1.** Quantification analysis of CD11b^+^ myeloid cells, M-MDSCs and G-MDSCs.

**Figure S2.** (A) Detection of CD3^+^ cells by flow cytometry. (B) Detection the proportion of CD4^+^, CD8^+^ T cells and Foxp3^+^ Tregs in skin allografts. (C) Induced-Tregs by M-MDSC with or without CHBP induction *in vitro*.

**Figure S3.** (A) Protein-protein interaction of different RNA expression in M-MDSCs with or without CHBP treatment *in vitro* by RNA-seq. (B) Different protein expression of M-MDSCs with or without CHBP treatment *in vitro* by protein array. (C) Biological process enrichment of protein array. (D) KEGG pathway analyses of protein array.
