# Supplementary figures and images for: CHBP induces stronger immunosuppressive CD127+ M-MDSC via erythropoietin receptor
Source: Cell Death Dis. 2021 Feb 12;12(2):177. doi: 10.1038/s41419-021-03448-7 (PMC7881243; doi:10.1038/s41419-021-03448-7)

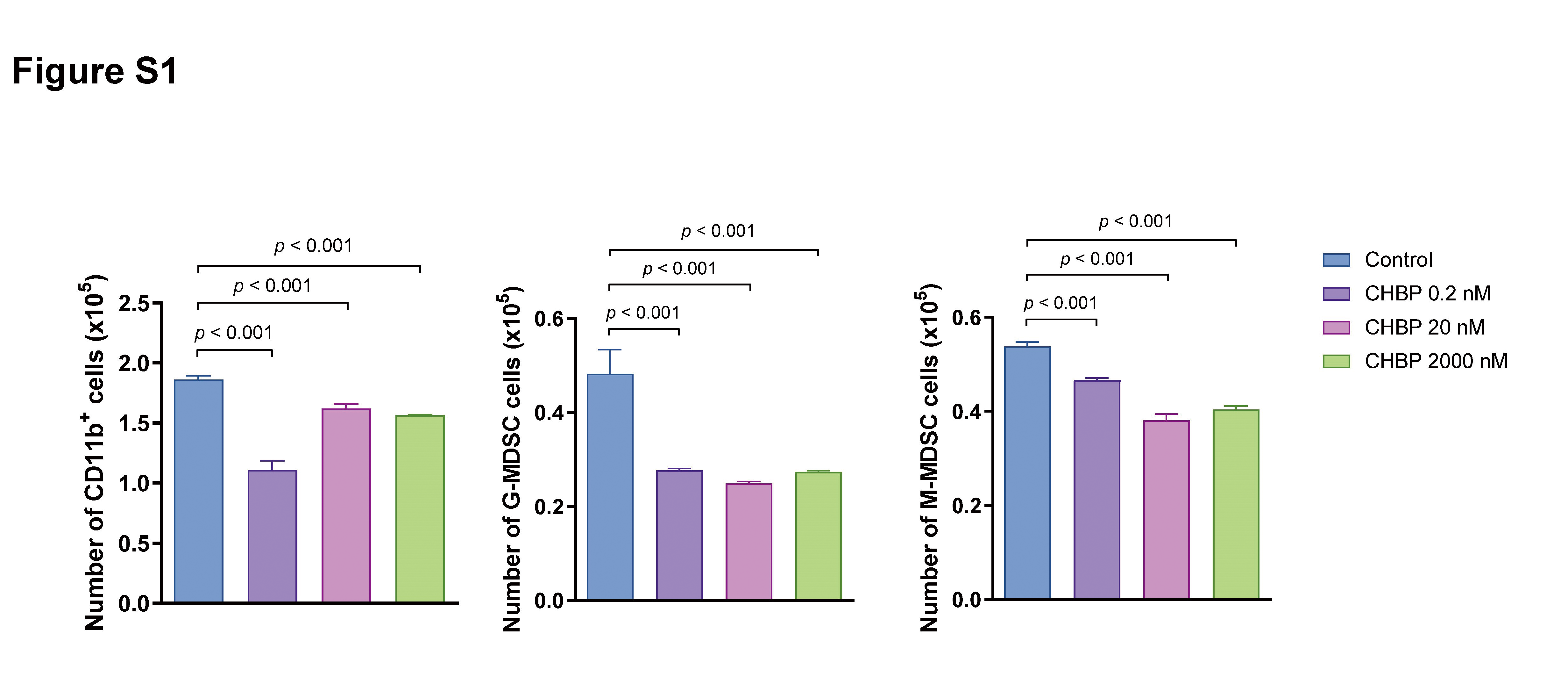

Supplement: Supplementary file 2 — Figure S1 [file 41419_2021_3448_MOESM2_ESM.png]

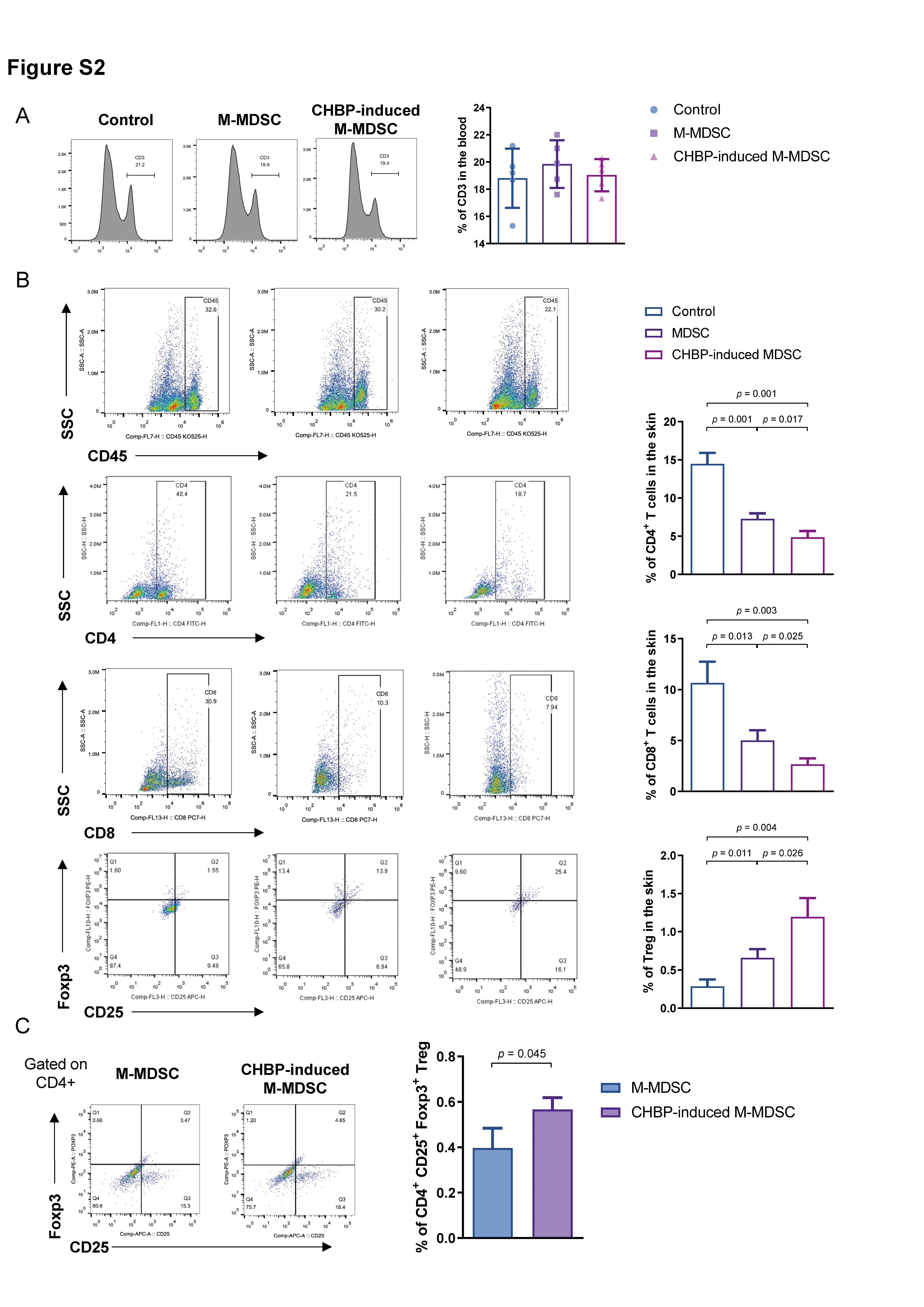

Supplement: Supplementary file 3 — Figure S2 [file 41419_2021_3448_MOESM3_ESM.png]

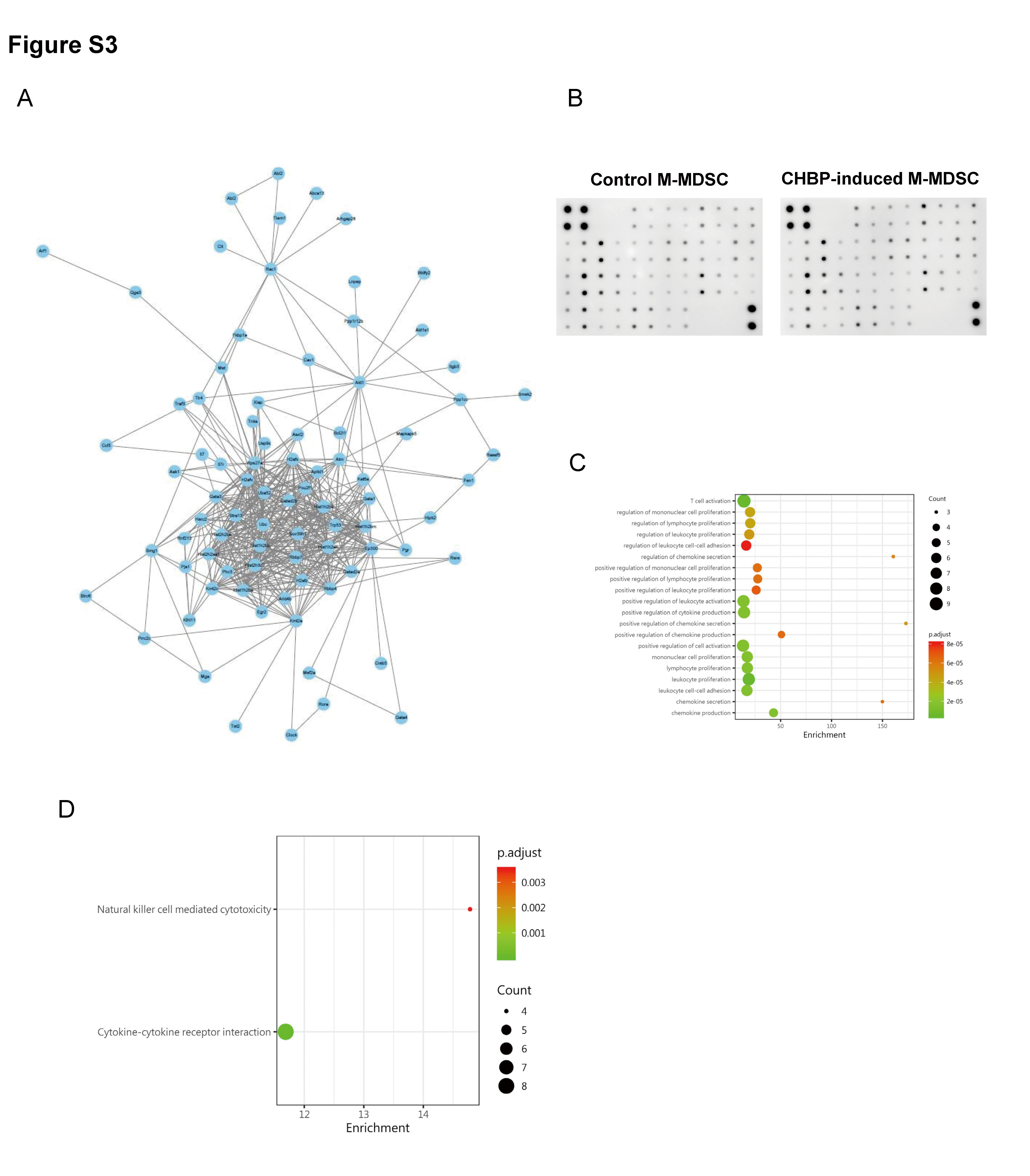

Supplement: Supplementary file 4 — Figure S3 [file 41419_2021_3448_MOESM4_ESM.png]
