# Supplementary material for: CHBP induces stronger immunosuppressive CD127+ M-MDSC via erythropoietin receptor
Source: Cell Death Dis. 2021 Feb 12;12(2):177. doi: 10.1038/s41419-021-03448-7 (PMC7881243; doi:10.1038/s41419-021-03448-7)
Supplement: Supplementary file 5 — Table S1 [file 41419_2021_3448_MOESM5_ESM.docx]

**Table S1. Primers sequence**

| **Genes** | **Forward primer** | **Reverse primer** |
| --- | --- | --- |
| **GAPDH** | 5’-GACTTCAACAGCAACTCCCAC-3’ | 5’-TCCACCACCCTGTTGCTGTA-3’ |
| **iNOS** | 5’-CACCAAGCTGAACTTGAGCG-3’ | 5’-CGTGGCTTTGGGCTCCTC-3’ |
| **Arg-1** | 5’-CCAGAAGAATGGAAGAGTCAGTGT-3’ | 5’-GCAGATATGCAGGGAGTCACC-3’ |
